# Role of integrins in the periodontal ligament: organizers and facilitators

**DOI:** 10.1111/prd.12027

**Published:** 2013-08-11

**Authors:** Malgorzata Barczyk, Anne Isine Bolstad, Donald Gullberg

Integrins are cell-surface receptors that connect cells to the collagen-rich mechanically stressed periodontal ligament microenvironment. In the vertebrate body the periodontal ligament is the tissue with the highest turnover of collagen. Three major groups of integrins are of particular interest in the periodontal ligament: (i) I-domain-containing collagen-binding integrins; (ii) non-I-domain integrins linked via ‘bridging’ molecules to collagen (we suggest the name ‘COLINBRI’ (collagen-integrin-bridging) for these bridging molecules); and (iii) transforming growth factor-beta activating integrins.

Limited knowledge exists regarding the role of integrins in the periodontal ligament during development and in disease. One exception to this is the collagen-binding integrin, α11β1, which has been shown, in a genetic mouse model, to have a role during mouse incisor tooth eruption. Integrins with similar roles in molar periodontal ligament fibroblasts have not been described, and limited information is available on the role of α11β1 in human periodontium. αvβ3 integrin binds to the COLINBRI protein, periostin, and to the microfibril protein, fibrillin-1. More detailed studies of this integrin are predicted to yield interesting results in the years to come. Essentially nothing is known about the role of transforming growth factor-beta activating integrins, such as α8β1, αvβ5 and αvβ8, on periodontal ligament fibroblasts or their potential contribution to periodontal ligament dynamics and COLINBRI binding.

In this article we will discuss the potential roles of integrins in periodontal ligament fibroblasts and periodontal ligament stem cells. We suggest that the major roles of integrins on mesenchymal periodontal ligament cells are to act as organizers of the matrix and as facilitators of tissue regeneration.

## The cells of the periodontal ligament

**Fig 1:**
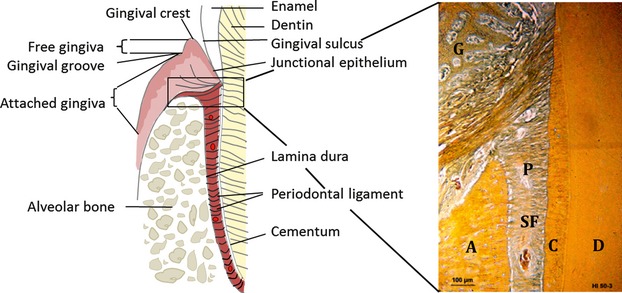
Structure of the periodontium. A, alveolar bone; C, cementum; D, dentin; G, gingiva; P, periodontal ligament; SF, Sharpey’s fibers. Courtesy of Knut A. Selvig.

The periodontium can be considered as an organ composed of hard tissues (cementum and alveolar bone) and vascularized soft tissues (gingiva and periodontal ligament) (Fig. 1). The extracellular matrix of these tissues is composed of members of the collagen family, proteoglycans and a heterogeneous set of glycoproteins (119).

The periodontal ligament is the only ligament in the body that connects two distinct hard tissues. It is a fibrous, complex, soft connective tissue that attaches the tooth root to the inner wall of the alveolar bone. The width of the periodontal ligament in human molars ranges from 0.15–0.38 mm, with the thinnest part around the middle third of the root. The corresponding values for mouse molar periodontal ligament are 0.1–0.13 mm (91). The periodontal ligament thickness decreases with age. It is functionally important for tooth support, and for allowing the teeth to withstand the forces generated during mastication.

Another important function of the periodontal ligament is to regulate alveolar bone volume and to serve as a cell reservoir for tissue homeostasis and regeneration (89). The periodontal ligament also acts as a sensory organ necessary for the proper positioning of the jaws during mastication. The periodontal ligament has very high adaptability to rapid changes in applied forces and capacity to maintain its width (90). This ability is an important measure of the periodontal ligament homeostasis. The alveolar bone is constantly remodeled in response to changes in the tooth micro-movement generated during mastication.

Periodontal ligament fibroblasts are the dominant cell population in the periodontal ligament. Other cell types include the epithelial cell rests of Malassez, monocytes, macrophages, endothelial cells and stem/progenitor cells. Periodontal ligament fibroblasts originate from ectomesenchymal cranial neural crest cells. The delicate balance of different transcription factors in regulating formation of molars and incisors in the maxilla and the mandible, and the important interaction between mesenchyme and dental epithelium during tooth formation, is known in some detail (145), but downstream targets during these steps, such as integrin-guided cell migration, are largely unknown. The fibroblasts in the periodontal ligament are a heterogeneous population (88), both within the same tooth and between different teeth. It is not known how this heterogeneity arises, but it is possible that similar transcriptional mechanisms are used as those operative when tooth identity is established. Compared with other fibroblasts, periodontal ligament fibroblasts are unique in that they possess the capacity to differentiate into cementoblasts and osteoblasts (90, 121). This osteogenic differentiation capacity is reflected in that periodontal ligament fibroblasts *in vivo* possess alkaline phosphatase activity (122). In the rodent incisor periodontal ligament, variations in the alkaline phosphatase activity have been reported between different subsets of periodontal ligament fibroblasts: periodontal ligament fibroblasts toward the tooth side express low alkaline phosphatase activity and periodontal ligament fibroblasts toward the bone side express high alkaline phosphatase activity (122). This further underscores the heterogeneity of fibroblasts in the periodontal ligament.

The periodontal ligament fibroblasts are characterized by a high rate of collagen turnover (134), which occurs by simultaneous synthesis and degradation of collagen fibrils. This aspect of the periodontal ligament cell biology is discussed more extensively in a later section on integrins.

In addition to the multipotent periodontal ligament fibroblasts, the periodontal ligament contains a low level of periodontal ligament stem cells, which also originate from the ectomesenchymal cranial neural crest cells and which possess the capacity to differentiate into periodontal ligament fibroblasts, cementoblasts and osteoblasts (11, 55, 135).

The distinction between periodontal ligament stem cell and periodontal ligament fibroblast populations in the periodontal ligament is therefore delicate. A major difference is that the stem cells are normally quiescent and only activated during tissue damage. The elements of gingival connective tissue originate from oral mucosa connective tissue. The gingival fibroblasts are of mesenchymal origin and are important for development, reorganization and regeneration of gingival connective tissue (12).

## The extracellular matrix of the periodontal ligament

As the periodontium includes gingiva, root cementum, periodontal ligament and alveolar bone it is important to distinguish between periodontium and periodontal ligament when discussing extracellular matrix molecules and integrins. In this review we will mainly focus on the periodontal ligament and the periodontal ligament fibroblasts.

### Collagens in the periodontal ligament

The extracellular matrix of the periodontal ligament contains collagenous and noncollagenous fibers. The majority of collagens in the periodontal ligament are organized in fiber bundles termed principal fibers. The extremities of collagen fiber bundles are embedded in the alveolar bone and the cementum of the root, and form mineralized Sharpey’s fibers. The major collagen types of the periodontium are collagen I and collagen III. Other collagens found in the periodontal ligament are collagens V, VI, XII and XIV (61, 124). Individual fibrils are continuously remodeled by fibroblasts whereas the overall fiber mesh maintains its structure and function. The expression of the remaining members of the collagen family (a total of 28 members (117)) is poorly defined with regard to the periodontal ligament.

The collagens of the periodontal ligament, which have been defined in some detail with regard to their cell-adhesive regions, are collagen I and collagen III. Using the so-called collagen toolkits and cells with different set-ups of collagen receptors, systematic studies of cell-adhesive regions have been conducted using overlapping peptides (40, 49). Collagens III and V form heterotypic fibers with collagen I and can regulate fibril diameter, whereas collagen VI associates with the microfibrillar system (39). Collagen XII is a fibril-associated collagen with interrupted helices (FACIT collagen) and as such mediates interactions between collagen fibrils. Genetic evidence for the role of collagens in the periodontal ligament exists but is relatively scarce.

Surprisingly, no periodontal ligament phenotype has been described in patients with Ehlers–Danlos syndrome with genetically characterized mutations affecting collagen (I/III/V) synthesis/assembly. However, in the uncharacterized form of Ehlers–Danlos syndrome type VIII, periodontitis is a typical feature (116). It will be interesting to identify the gene that is mutated in Ehlers–Danlos syndrome type VIII and determine how it contributes to collagen matrix integrity (Table 1). A mouse model for a mutated and overexpressed collagen XII exists in which disturbed periodontal ligament organization is observed, supporting role in organization of collagen XII in the periodontal ligament (115). More recently, a mouse deficient in collagen XII has been generated (58). The characteristic feature of this collagen XII^−/−^ mouse is a bone phenotype; no tooth-related defects were described in this model, suggesting that the transgenic overexpressing phenotype might have limited physiological relevance.

**Table 1:** Effect of extracellular matrix mutations in the periodontium

Ligand	Periodontal ligament	Human disease	Mouse model	Periodontal ligament phenotype	Reference(s)
Collagen					
Collagen I	✓	Ehlers–Danlos syndrome type VIIa/b		^−^	(1)
Collagen XII	✓		*Col12a1*^−/−^ *MXIINC3(-) TG*	ND +	(58, 115)
Proteoglycans					
Decorin	✓	Stromal corneal dystrophy	*Dcn*^−/−^	+ (mouse) ND (human)	(23, 33)
Lumican	✓		*Lum^−/−^*	+	(28)
Fibromodulin	✓		*Fmod*^−/−^	+	(140)
Glycoproteins					
Fibrillin-1	✓	Marfan syndrome type I	*Fbn1^−/−^ Fbn1^Tsk^*	+	(108, 133)
SPARC	✓		*Sparc*^−/−^	+	(102)
Osteopontin	✓		*Spp1^−/−^*	^−^	(77)
Periostin	✓		*Postn^−/−^*	+	(120)
Transforming growth factor beta-induced	✓	Granular corneal dystrophy	*Tgfbi^−/−^*	ND	(66, 167)
Tenascin-W	✓		^−^	ND	(100)
Gene locus: 12p13	✓	Ehlers–Danlos syndrome type VIII	^−^	+	(16)

+ present

− none

ND, not determined.

### Proteoglycans in the periodontal ligament

In recent years the role of proteoglycans in tissue-specific collagen reorganization has attracted increasing interest. Data from decorin-deficient animals have convincingly demonstrated an important role for decorin in collagen organization of the periodontal ligament (48). Separate studies using knockout mouse models have suggested that lumican and fibromodulin are also involved in supramolecular organization of periodontal ligament collagen (87). Less is known about the role of other small leucine-rich proteoglycans in the periodontal ligament. In an attempt to generate a database for the periome, periodontal ligament-enriched mRNAs were found to include some small leucine-rich proteoglycans (100), although the expression and tissue distribution was not studied in any detail in that study.

### Multiadhesive glycoproteins of the periodontal ligament

Fibrillin-1 is part of the oxytalan fibers in the periodontal ligament. Elastic oxytalan fibers identified within the periodontal ligament are described as a three-dimensional meshwork surrounding the root and terminating in the apical complex of arteries, veins and lymphatic vessels. It is believed that oxytalan fibers regulate vascular flow to the tooth. These fibers contain fibrillin-1 and elastin, but in the human periodontal ligament no elastin is present in these fibers (60, 130). Collagen VI localizes to this microfibrillar network (39). Marfan syndrome patients with fibrillin-1 mutations have a tooth phenotype, and a hypomorphic fibrillin-1 mouse model displays a periodontal ligament phenotype (125, 130, 137, 138) (Table 1). As fibrillin-1 in other tissues has a role in modulating transforming growth factor-beta activation, it is possible that it plays a similar role in the periodontal ligament (138).

The protein, periostin, is highly expressed in molar and incisor periodontal ligament (63) and can interact with collagen I (103). A detailed study of how periostin affects collagen stiffness suggests that periostin dimers crosslink the collagen matrix (131). Periostin-mutant mice have an incisor-eruption defect characterized by a disorganized periodontal ligament (63). Periostin, by being able to bind both collagen I and cells, is one of the most interesting extracellular matrix molecules in the periodontal ligament.

Transforming growth factor beta-induced (previously called big-H3) is related to periostin and was, in one study, reported to inhibit the mineralization of periodontal ligament cells *in vitro* (105), but later work has focused on its role in corneal dystrophy (66) and in tumor biology (157, 167). Recently, inactivation of the gene was reported to result in reduced periostal bone formation, but a defect in the periodontal ligament was not reported (162). Given the close relationship of transforming growth factor-BI to periostin, it might be worthwhile to revisit this molecule.

Tenascin-C has a localized distribution toward the attachment zone to bone in the periodontium and appears not to be expressed throughout periodontal ligament (166). In a systematic study of periodontal ligament-expressed genes, tenascin-W (also called tenascin-N) was reported to be highly expressed throughout both molar and incisor periodontal ligament matrix (100, 151). Tenascin-W appears to be a good biomarker for the periodontal ligament, but currently the information about the biology of this molecule in the periodontal ligament is scarce.

Several markers exist for the mineralizing cementum, including bone sialoprotein, osteocalcin, osteopontin and secreted-protein-acidic-and-rich-in-cysteine. However, osteopontin and secreted-protein-acidic-and-rich-in-cysteine are not restricted to mineralizing cells as they are also expressed by periodontal ligament fibroblasts. Defects in periodontal ligament collagen organization have been noted in older secreted-protein-acidic-and-rich-in-cysteine^−/−^ mice, which was suggested to reflect a secreted-protein-acidic-and-rich-in-cysteine-dependent regulation of collagen turnover by periodontal ligament fibroblasts (148). Osteopontin and bone sialoprotein belong to the small integrin-binding ligand N-linked glycoproteins (SIBLINGs) (17). Osteopontin in the periodontal ligament was suggested to prevent mineralization, but no changes in mineralization have been observed in the periodontal ligament of osteopontin^−/−^ mice (31).

To summarize, for some, but not all, structural extracellular matrix components, mutations in the genes encoding these components cause structural defects in the periodontal ligament. Furthermore, additional secondary cellular effects, owing to disturbed periodontal ligament tissue-organization defects, might arise. Theoretically, if mutations/defects affect integrin binding, additional integrin-related cell structural and signaling-related effects might arise. Although collagens I/III are the most abundant components of the extracellular matrix, many unanswered questions, relating to how cells interact with the collagen network, remain. It seems likely that both direct interactions, via collagen-binding integrins, and indirect integrin interactions, mediated via collagen-binding bridging molecules, exist to secure proper periodontal ligament fibroblast anchorage in the periodontal ligament.

Two periodontal ligament extracellular matrix components that stand out as potentially having more intricate effects are fibrillin-1 and periostin. Fibrillin-1 might attenuate transforming growth factor-beta activation within the tissue. Periostin, by binding multiple αv-integrins and collagen I, can serve as an important bridging molecule in the periodontal ligament tissue. Considering the emerging realization of cell heterogeneity within the periodontal ligament tissue, it is currently unclear what the relative contributions of minor progenitor/stem cells are to the extracellular matrix synthesis of the periodontal ligament. In the future it will be important to determine if stem cells produce low-abundant extracellular matrix components locally, which then combine to form periodontal ligament stem-cell niches with special properties.

## Integrins in the periodontal ligament

**Fig 2:**
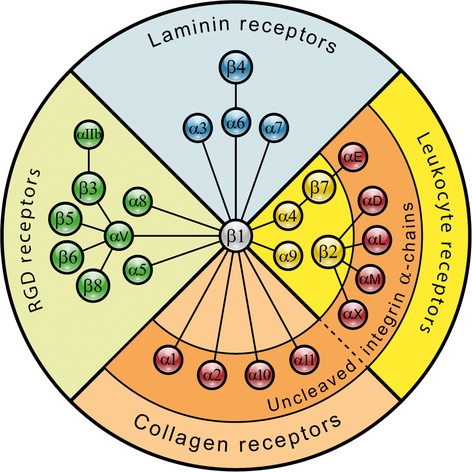
Schematic illustration of the vertebrate integrin family.

In vertebrates the integrin family is composed of 18 α-subunits and 8 β-subunits that can assemble into 24 different heterodimers (6) (Fig. 2).

### Non-I domain α-subunits

The α-subunit is composed of a seven-bladed β-propeller that forms a head domain which is connected to a thigh domain, a calf-1 domain and a calf-2 domain, together forming the leg structure that supports the integrin (54, 106). The ligand-binding site forms in a region at the intersection of the integrin α-chain β-propeller and the βI domain, with the α-chain being central in determining ligand specificity.

### I-domain α-subunits

Nine of the integrin α-chains contain an I domain (also called the A domain), which is composed of approximately 200 amino acids and inserted between blades 2 and 3 in the β-propeller (72). Integrins with an αI domain bind ligands via the αI domain, but because this ligand binding causes distinct conformational changes in the I domain, this, in turn, affects the conformation of the β-subunit (83). The αI domain is present in chordate and vertebrate integrins but is absent in invertebrates (59). The αI domain is present in the β2 integrin subgroup of integrins, in the collagen-binding integrins belonging to the β1 subfamily (α1, α2, α10 and α11) and in the αE integrin chain forming the αEβ7 heterodimer. The I domain assumes a Rossman fold, with five β-strands forming a β-sheet surrounded by seven α-helices. Ligand binding occurs via a coordinating Mg^2+^ ion in the so-called metal-ion-dependent adhesion site (74). The αI domains with the capacity to interact with collagens, in addition also contain a so-called αC helix (37), which is suggested to play a role in collagen binding. αI domain-containing integrins show fairly high homology in the αI domains, but the α-chain cytoplasmic domains are highly divergent, only sharing the GFFKR sequence, or even the core GFFXR sequence, in the membrane proximal region. Few specific interactions of the α-chains have been described, except for the paxillin interaction with α4 and α9 cytoplasmic tails (79, 80) regulating cell spreading and cell migration. More recently, Mena binding to the α5 cytoplasmic tail has been shown to regulate fibronectin fibrillogenesis (47).

### Integrin β-subunits

The extracellular part of the β-subunit contains a plexin, semapahorin, integrin (PSI) domain, a hybrid domain, a βI domain (74), four cysteine-rich epidermal growth factor repeats and a β-tail domain. The βI domain contains an Mg^2+^ coordinating metal-ion-dependent adhesion site and a site adjacent to the metal-ion-dependent adhesion site that binds an inhibitory Ca^2+^ ion. It is this site adjacent to the metal-ion-dependent adhesion site that binds an Mn^2+^ ion, leading to a conformation change resulting in an active form of the integrin (54). The β-integrin chains share homology in the cytoplasmic tail, with NPX/Y motifs able to bind proteins containing Phosphotyrosine binding domains. In recent years several proteins have been found to interact with the β-subunit (75).

Some key proteins seem to be essential for inside-out integrin activation. Detailed studies of the interacting regions in the αΙΙb- and β3-subunit transmembrane domains suggest a model for conformation-mediated changes over the membrane (73). Talins 1 and 2 and kindlins 1–3 seem to act synergistically to activate integrins by binding to integrin β-subunit tails (71, 128, 142), whereas filamin A negatively regulates activation (62). During activation the salt bridges with the α-subunit, which normally keep the integrin in the inactive conformation, are broken. Migfilin is another molecular switch which, by blocking the integrin-binding region of filamins (56), can regulate integrin activation. Integrin-linked kinase (52) and focal adhesion kinase (93), best known for taking part in outside-in signaling, have also been shown to affect integrin activation via inside-out signaling.

### Prototypic integrin ligands and recognition sequences

The list of integrin ligands is long (53, 59) and includes the major constituents of the extracellular matrix. The prototypic integrin ligand, fibronectin, contains the amino-acid sequence RGDS at the apex of the flexible loop connecting two β-strands in the 10th fibronectin type III repeat (35). The RGD sequence is also present in vitronectin, fibrinogen, and the latency-associated peptide complex part of inactive transforming growth factor-beta and in many other extracellular matrix proteins (53). Elegant studies have shown that the epithelial αvβ6 and αvβ8 integrins, by binding RGD in latency-associated peptide, are the major integrins that activate transforming growth factor-beta *in vivo*, either by allosteric changes in the transforming growth factor-beta/latency-associated peptide (αvβ6) complex or by inducing matrix metalloproteinase-14 and causing proteolytic release of transforming growth factor-beta (αvβ8) (3, 97, 98). More recently, a mechanical strain-dependent contribution of myofibroblast β1 integrins to transforming growth factor-beta activation has been demonstrated (159). In addition, the integrins αvβ3 (159), αvβ5 (4) and α8β1 (82) can all bind latency-associated peptide, but the extent to which they can activate the transforming growth factor-beta/latency-associated peptide complex in different cellular contexts remains to be determined.

RGD sequences present in triple helical fibrillar collagen sequences are normally not available for fibronectin receptors in the native fibrillar collagen (34, 46), only becoming available in denatured collagen I. Instead, the collagen-binding integrins recognize the triple-helical GFOGER sequence (67), or variants thereof, in native collagens. An assembled database on collagen I-binding sites and mutations has enabled the construction of a model where the GFOGER site is present in a cell-interactive domain that is suggested to be exposed once per microfibril unit (each microfibrillar unit is composed of five collagen triple helix monomers), allowing clustering of integrins (141). However, a number of components in the fibrillar matrix might influence the availability of these sites.

**Fig 3:**
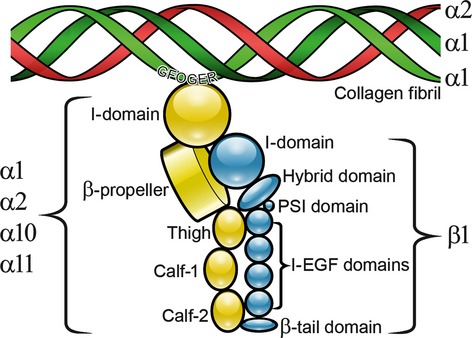
Schematic illustration of the interaction of collagen-binding integrins with fibrillar collagens. The integrin β1-subunit is in blue and the integrin α-chain is in yellow. PSI, Plexin, semaphorin and integrin; EGF, Epidermal growth factor.

Certain variants of the GFOGER sequence might show specificity for the different collagen-binding integrins, as indicated by a recent finding showing that the bacterial collagen-like peptide, sscl, with the active binding-site GLPGER, is preferred by α11β1-expressing cells over α2β1-expressing cells (27). More recently, GFOGEN has been identified as a collagen sequence recognized by α1β1 (49, 129) (Fig. 3, Table 2).

**Table 2:** Collagen-binding integrins and their ligand-recognition motifs

Integrin	Collagen specificity	Recognition sequence specificity	References
α1β1	Collagen I > Collagen IV	GFOGER (I, II, IV and more) > GVOGEA (collagen II) GLOGEN (collagen III) GFPGEN (Scl2)	(49, 68, 129, 132 152)
α2β1	Collagen I > Collagen IV	GFOGER (I, II, IV and more) > GLPGER (rScl1) GFPGER (Scl2)	(27, 129, 146 152)
α10β1	Collagen IV > Collagen I Collagen II Collagen III	GFOGER (I, II, IV and more)	(26, 129)
α11β1	Collagen I > Collagen IV	GFOGER (I, II, IV and more) > GLPGER (rScl1)	(27, 146, 165)

> greater than.

Although the list of integrin ligands is long and we are a decade into the postgenomic era, new integrin ligands are still being characterized. Recently, the widely expressed extracellular matrix protein, polydom, was identified as a high-affinity integrin α9β1 ligand (127).

### Integrins as mechanical links

The first function established for integrins was their function as links between the extracellular matrix and the cytoskeleton. For a majority of integrins, the linkage is to the actin cytoskeleton (44). Some of the components in this mechanical linkage, such as talin, play a dual role and also take part in activating integrins in an inside-out signaling mechanism (93). A new dimension of integrins as mechanical links has come with the realization that integrins can act as mechanosensors and generate signals that affect cell physiology via complex intracellular signaling mechanisms, including autocrine and paracrine mechanisms (30, 78, 94, 95, 168). Mechanical tension can also increase integrin affinity (22).

### Integrins as signaling receptors

Integrins are bidirectional signaling receptors involved in outside-in and inside-out signaling. The inside-out signaling mainly acts to bring the integrin into the active conformation. Talin, kindlins, filamins, migfilin, focal adhesion kinase and integrin-linked kinase (52) can all regulate integrin activation. Upon ligand binding, integrins undergo conformation changes leading to outside-in signaling. This activates signaling events that vary depending on the cellular processes affected. Typical integrin-induced signaling includes focal adhesion kinase phosphorylation and mitogen-activated protein kinase activation. In general the signaling is cell specific, and the end cellular effect then depends on what other signaling receptors and signaling systems are available in the cell (5, 42, 71, 76, 84). Limited knowledge exists on integrin signaling in the periodontal ligament.

### Integrins as regulators of matrix metalloproteinases and cathepsins

A number of studies in fibroblasts and in other cell types have indicated that integrins can regulate matrix metalloproteinase synthesis. Mice deficient in matrix metalloproteinase-14 display disturbed eruption of molar teeth (10, 15). As matrix metalloproteinase-14^−/−^ mice die before reaching adulthood, the role of matrix metalloproteinase-14 in continuous incisor eruption cannot be evaluated in this model. Matrix metalloproteinase-8 and matrix metalloproteinase-13 have also been studied in the context of tooth eruption and tooth movement (143, 149). Both enzymes are dynamically regulated when teeth are subjected to pressure loading, suggesting an active role of these enzymes in periodontal ligament matrix remodeling. Surprisingly however, no periodontal ligament phenotypes have been described for mice deficient in these matrix metalloproteinases. A regulatory role for integrins in matrix metalloproteinase synthesis is well documented. The collagen-binding integrin, α1β1, regulates matrix metalloproteinase-9 (113); α2β1 has been reported to regulate matrix metalloproteinase-1 and matrix metalloproteinase-13 (70, 114, 118); and, in mouse embryonic fibroblasts and incisor periodontal ligament fibroblasts, α11β1 appears to regulate both matrix metalloproteinase-13 and cathepsin K (7, 110)

Cathepsins constitute a family of enzymes involved in lysosomal degradation (153). Humans deficient in cathepsin K have a bone defect as a result of defective osteoclast function (32, 160). Data are emerging that cathepsin K also has important functions in cell types other than osteoclasts, including fibroblasts (163, 164). Recently, a complex cross-talk with another family member, cathepsin S, has been shown to regulate cathepsin K (9). A different member of the cathepsin family, cathepsin C, has been attributed to another role. Mutations in the *CTSC* gene, which encodes cathepsin C, have been identified as the underlying genetic defect in Haim–Munk syndrome and in the clinically related disorder, Papillon–Lefèvre syndrome, both of which are characterized by premature and severe periodontal disease (50).

**Fig 4:**
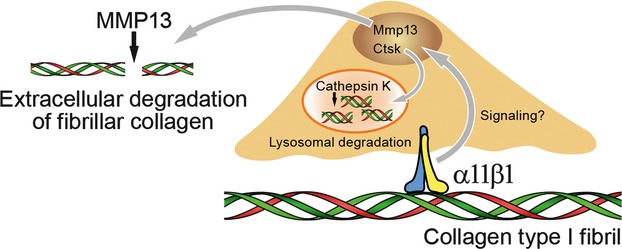
α11β1 integrin as a master regulator and coordinator of lysosomal cathepsin K (Ctsk)-mediated and extracellular matrix metalloproteinase-13 (MMP-13)-mediated proteolysis of collagens. *Ctsk*, cathepsin K gene.

We suggest that the recently described α11 integrin-dependent regulation of matrix metalloproteinase-13 and cathepsin K offers a unique mechanism for coordinated extracellular and intracellular collagen proteolysis (Fig. 4).

## Role of integrins in the periodontal ligament

Regulation of the reorganization of extracellular matrix and the role of different cell types in the periodontal ligament is still poorly understood at the molecular level. The periodontal ligament fibroblasts interact with the extracellular matrix via integrin binding of different collagenous and noncollagenous substrates. Interaction of integrins with the extracellular matrix extracellularly and with cytoskeletal components intracellularly are considered to be force-transducing elements in fibroblasts (2).

The majority of studies on periodontal ligament integrins have been performed on cultured human molar periodontal ligament fibroblasts, and little is known about periodontal ligament integrins at the tissue level. Cultured human molar periodontal ligament fibroblasts express mRNAs for multiple integrins but in the absence of protein data, it is unclear what this data means (24, 69, 107). Surprisingly few studies have thus dissected out clean periodontal ligament tissue. For a rigorous investigation of integrins in periodontal ligament fibroblasts, the following analyses are needed: (i) integrin RNA expression in specific cell types *in vivo* and confirmed by *in-situ* hybridization; (ii) western blotting, to confirm the presence of protein; and (iii) immunostaining, to localize specific integrin subunits within the periodontium. For isolated periodontal ligament cells a limited number of studies have performed functional studies using integrin-blocking antibodies. To our knowledge the α11^−/−^ mouse is the only integrin-deficient mouse model described to have a periodontal ligament phenotype (110).

It is important to remember that for periodontal ligament fibroblasts, as with all fibroblasts, the integrin repertoire changes upon cell culture. Furthermore, because periodontal ligament fibroblasts have the capacity to differentiate into other cell types, they display more plasticity compared with normal adult tissue fibroblasts. Primary periodontal ligament fibroblasts, with this plasticity feature, might be more prone to undergo changes in gene expression with time in culture, including integrin expression. Factors to consider as modulators of integrin expression include: stiffness of the culture surface; two-dimensional versus three-dimensional culture; serum content of media; and epigenetic changes. Function-blocking antibodies, as well as antibodies suitable for immunostaining, are available, and it should be relatively easy to sort out more systematically the relevant integrins on periodontal ligament cells *in vivo*. For determination of integrin function *in vitro*, whenever possible, freshly isolated cells should be compared with passaged cells. We will now discuss integrins in the context of (i) noncollagen binding-integrins linked to collagen via ‘bridging’ molecules, (ii) transforming growth factor-beta activating integrins and (iii) collagen-binding integrins; we will also discuss, in more detail, the α11β1 integrin.

### Noncollagen-binding integrins indirectly linked to collagen via bridging ligands (COLINBRIs)

Before the isolation of collagen receptors, a widely held belief in the cell-adhesion field claimed that cells did not interact directly with collagen, but via ‘nectins’ (65, 144). We now know that cells do interact directly with native collagens via the four collagen-binding integrins (111). A provocative study suggests that the fibrillar organization of collagen itself in tissues makes the cell-binding sites inaccessible to cells (109). Therefore, in adult tissues, the collagen-binding integrins would have minimal access, and limited proteolysis of collagen would be required for integrin access to the cell-binding sites in fibrillar collagen. This would also suggest that collagen-binding integrins are mainly involved in establishing immature collagen matrices during development and that they perform similar collagen assembly/organization roles in tissues with high turnover (like the periodontal ligament). It would also imply that collagen receptors mainly have access to collagens during tissue repair and regeneration events. This is a very interesting concept, which, however, might be difficult to address experimentally *in vivo*. With accumulating data on the complexity of the interactions in the extracellular matrix demonstrating an increasing diversity of extracellular matrix interactions, not only between extracellular matrix components, but also with growth factors/cytokines and matrix metalloproteinases, it is time to revisit the ‘nectin’ concept. COLINBRI molecules comprise a class of molecules with the potential to bridge the binding of non-I domain integrins, such as fibronectin receptors and vitronectin receptors, to the collagen matrix.

Fibronectin is a classical COLINBRI molecule, and fibronectin binds both native and denatured collagens, although the binding to denatured collagen is of higher affinity (38, 123).

α5β1 integrin is the classical fibronectin-binding integrin, is present on cultured periodontal ligament fibroblasts (57, 101, 154) and has been immunolocalized to periodontal ligament fibroblasts as well as to other cell types in primate periodontium (136).

Vitronectin has been reported to be abundantly expressed in primate molar periodontal ligament. Vitronectin binds native collagen I (96) and thus might be an important COLINBRI molecule in the periodontal ligament (136). αvβ3 is perhaps best known for its role on osteoclasts (92), but αvβ3 has also been detected on primate molar periodontal ligament fibroblasts *in vivo* (136) and on cultured human molar periodontal ligament fibroblasts (150, 154, 156) where it can bind vitronectin.

Periostin is clearly an important COLINBRI molecule, binding both integrins and collagens and having a structural role in the periodontal ligament (120, 131). In one study it was shown that αvβ3-mediated binding of human molar periodontal ligament fibroblasts to periostin induced matrix metalloproteinase-2 synthesis (156).

*In-vitro* studies have furthermore shown that human molar periodontal ligament fibroblasts interact with ‘processed’ collagen V via αvβ3 integrin, but it is possible that other RGD-recognizing integrins can also mediate interactions with denatured collagen V (19). The *in-vivo* relevance of αvβ3 binding to unfolded collagen V is unclear because, under normal physiological conditions, collagen V in heterotypic fibrils with collagen I might not be accessible to cells. However, in pathological conditions with high collagen turnover this interaction might take place once the RGD sites are exposed in denatured collagen V.

The *in-vitro* data on cultured periodontal ligament cells suggest that closer examination of a possible tooth periodontal ligament phenotype in αv-deficient mice is warranted. As αv integrins have the capacity to mediate the effects of multiple ligands present in the periodontal ligament, possible compensation mechanisms that might be operational when αv integrins are lacking are likely to be complex. It will therefore be important to determine which compensating integrin(s) is(are) induced and how these integrins compare with regard to specialized functions normally carried out by αvβ3.

### Transforming growth factor-β-activating/attenuating integrins in the periodontal ligament

Transforming growth factor-beta1 is present in the murine periodontal ligament (41), but the exact role that this multifunctional cytokine plays in periodontal ligament physiology is not clear. For quite some time now it has been known that certain integrins are essential in the activation of transforming growth factor-beta (99). Convincing genetic data have shown that a majority of the integrin-dependent activating effects can be traced back to the RGD sequence in latency-associated peptide (161). Owing to the short lifespan and inflammatory phenotype of the latency-associated peptide-mutated mice, it was not possible to challenge these mice and test the possible contribution of non-RGD-binding integrins to transforming growth factor-beta activation.

In the tooth context it has been reported that αvβ6 integrin-mediated transforming growth factor-beta1 activation in the junctional epithelium plays a protective role in inflammatory periodontal disease (45). β6-deficient mice develop classic symptoms of chronic periodontal disease. In a rat model, block of αvβ6 led to the appearance of the initial signs of periodontitis. αvβ6 integrin is also down-regulated in human periodontal disease (45).

However, little is known about transforming growth factor-beta-activating integrins in the periodontal ligament tissue itself. As mentioned above, the current paradigm is that only RGD-binding integrins are transforming growth factor-beta activating, either by binding latency-associated peptide and unfolding the transforming growth factor-beta/latency-associated peptide complex or by inducing matrix metalloproteinase-14 synthesis, which cleaves and activates the complex (85). RGD-binding integrins on periodontal ligament fibroblasts that potentially could carry out such a function include αvβ3, αvβ5 and αvβ8 (64, 158, 159). On other types of fibroblasts a contribution of β1 integrins to transforming growth factor-beta activation has also been shown (81), perhaps suggesting the involvement of latency-associated peptide in binding αvβ1 or α8β1 integrins.

Fibrillin-1 exists in fibrils independent from the collagen network. Data suggest that assembly of fibrillin-1-containing oxytalan fibers may be controlled by αvβ3 on periodontal ligament fibroblasts (150). αvβ3, by binding fibrillin-1, may also take part indirectly in transforming growth factor-β activity modulation. Further studies are needed to address these issues.

### Collagen-binding integrins in the periodontal ligament

The three collagen receptors that have been detected on periodontal ligament fibroblasts at the protein level are α1β1, α2β1 and α11β1. All of these integrins are present at various levels on a variety of cultured periodontal ligament fibroblasts, but only α2β1 and α11β1 have been shown, by immunohistochemistry, to be present in the periodontal ligament *in vivo*. Neither α1β1 nor α2β1 are restricted to fibroblasts but are, in other tissues, expressed in epithelial cells, capillaries and osteoblasts (111). However, α11β1 expression in the periodontium is, based on our current knowledge, restricted to ectomesenchymally derived periodontal ligament fibroblasts (112).

No systematic study of collagen-binding integrins on progenitors/stem cells in the periodontal ligament has been performed. α2β1 has been detected *in vivo*, by immunohistochemistry, in primate molar periodontal ligament fibroblasts (136) and is also abundantly expressed in cultured human molar periodontal ligament fibroblasts *in vitro*.

α2β1 is only sparsely expressed on cultured mouse incisor periodontal ligament fibroblasts.

The immortalized mouse molar periodontal ligament-L2 cell line expresses α1β1 as its only collagen receptor, but notably lacks α2β1 expression (7, 126). Mouse incisor periodontal ligament fibroblasts seem to express α11β1 as their major collagen receptor *in vivo* and *in vitro* (7). Interestingly, cultured mouse incisor periodontal ligament fibroblasts also express α1β1 and α2β1 upon culture.

To our knowledge, no studies have been performed on human incisor periodontal ligament fibroblasts. *In vivo*, α11β1 is prominent on periodontal ligament in mouse incisors but not on periodontal ligament in mouse molars. In human periodontal ligament cells, α11 is expressed in molar periodontal ligament tissue and on cultured adult molar fibroblasts (Table 3). At this time it is premature to say whether α2β1 is expressed at higher levels on human molar periodontal ligament fibroblasts compared with mouse molar periodontal ligament fibroblasts, and if α2β1 is more highly expressed on molar periodontal ligament fibroblasts compared with incisor periodontal ligament fibroblasts. This will have to await a more extensive characterization of α2β1 expression on human incisors and mouse molar periodontal ligament fibroblasts.

**Table 3:** Expression of collagen-binding integrins in mouse and primate periodontal ligament

Integrin chain	Molar periodontal ligament		Incisor periodontal ligament		Molar periodontal ligament fibroblasts		Incisor periodontal ligament fibroblasts	
	Primate	Murine	Primate	Murine	Primate	Murine	Primate	Murine
α1	ND	ND	ND	−	+	+	ND	+
α2	+	ND	ND	−	+	−	ND	+
α10	ND	ND	ND	−	−	−	ND	−
α11	+	−	ND	+++	++	−	ND	++

+ weak expression; ++, moderate expression; +++, strong expression;

− no expression; ND, not determined.

From a developmental point of view it will be interesting to determine how ectomesenchymal cells in different teeth show such a distinct expression of specific integrins. This might be related to epigenetic silencing mechanisms being receptive to different microenvironments. Recently, expression of the β8 integrin subunit was shown to be regulated by epigenetic mechanisms: whilst lung fibroblasts were found to express β8, dermal fibroblasts did not (86).

Relatively few functional studies of integrin–extracellular matrix interactions in periodontal ligament-derived cells have been conducted. *In vitro*, α1β1, α2β1 and α11β1 all contribute to cell attachment to monomeric collagens I and III, and cell migration on monomeric collagen I (8).

As α1β1 does not seem to interact with fibrillar collagen I (152), its contribution to collagen reorganization, as evaluated by collagen gel-contraction assays, is probably limited. Instead, α2β1 and α11β1 seem to fulfill this function on periodontal ligament fibroblasts. α2β1 and α11β1 integrins are known to enhance polymerization of collagens I and III, which indicates a role in collagen matrix assembly for these integrins (155). The role of assembling and contracting the collagen matrix, we call the organizer role. This role is central during development and serves to maintain tissue homeostasis. During the reorganization of collagen matrices, matrix metalloproteinases are regulated by integrins and are thought to facilitate the reorganization of collagen matrices (20, 70, 147).

In a recent study, periodontal ligament fibroblasts inside collagen gels were exposed to cigarette smoke (24). These exposed fibroblasts displayed a reduced ability to contract collagen lattices. Based on our current knowledge of integrin involvement in collagen-gel contraction this would correspond to reduced matrix metalloproteinase-facilitated integrin activity or reduced levels of collagen-binding integrins. When analyzing integrins and matrix metalloproteinases at the RNA level, increased levels of mRNAs encoding integrins and matrix metalloproteinases were noted, giving little mechanistic insight into the mechanism of reduced collagen-gel contraction. Future studies, using zymography combined with analyses of integrin function, integrin protein levels and integrin-dependent signaling events, are needed to gain insight into the mechanisms behind the reduced contractility of these cells.

We furthermore believe that the reorganiziation of the collagen matrix during periodontal ligament injury or disease is a very central role of periodontal ligament fibroblasts, involving dynamic regulation of matrix turnover, transforming growth factor-beta activation and collagen reorganization, and we call this property the facilitator role of periodontal ligament fibroblast integrins. This property may be important for periodontal ligament regeneration after injury.

## The collagen-binding α11β1 integrin

As discussed earlier in this review, the role of integrins in the periodontal ligament is a largely underexplored area. The only integrin shown to display a phenotype in the periodontal ligament in genetic knockout models is α11β1. We will now summarize the current knowledge of α11β1 in the context of the periodontal ligament.

### *In-vitro* functions of α11β1 in periodontal ligament fibroblasts

Antibodies that block α11β1-mediated cell attachment are still not available. To study the specific properties of α11β1 in a cellular context some other tools have been used, including α11β1-overexpressing cells (146) and mouse embryonic fibroblasts expressing or lacking α11β1 (110). Cell studies have shown that α11β1 prefers collagen I to collagen IV (146). Studies of α11 I domain binding to collagens have confirmed this finding and reinforced the impression that α11β1 displays similar collagen specificity to that of α2β1. The restricted distribution of α11β1 during human embryogenesis to regions of forming cartilage, bone, invertebral discs and cornea warrants studies of α11β1 binding to the collagens present at these sites. One interesting candidate is the transmembrane collagen XIII, previously reported to bind α1β1 (104), but which is also expressed in a pattern (139) compatible with it acting as a ligand for α11β1 in some tissues.

The collagen-binding integrins α2β1 and α11β1 (146) are both able to contract collagen matrices. This ability is likely to be important *in vivo* and to contribute to collagen organization during development. Similarly, during remodeling processes, such as wound healing, tissue regeneration and fibrosis, the reorganization of collagen matrices is likely to be driven by cells. The potential role of α11β1 in these processes remains to be determined.

α11β1 appears to be one of the receptors well suited for the task of mediating movement through a collagen-rich extracellular matrix. It will be important to determine if this function of α11β1 is more dominant in some cell types than in others. The finding that platelet-derived growth factor-BB and serum stimulate collagen-dependent chemotaxis in an integrin-specific manner (146), indicates that although cells express multiple collagen receptors, all of which are equally well suited for supporting cell migration, tissue-specific signals might preferentially engage a particular collagen-binding integrin for migration. It will be important to determine which collagen receptors are used for cell migration in various tissues.

### Physiological *in-vivo* function of α11β1, a lone collagen receptor on mouse incisor periodontal ligament fibroblasts

Comparison with the expression patterns of other collagen-binding integrins suggests that the expression of α11 partially overlaps with that of α2 and is complementary to that of α10 in many locations (112). Careful analyses of the phenotypes of mice deficient in individual collagen-binding integrins have revealed relatively mild phenotypes in unchallenged mutant mice (18, 29, 43, 51). The unchallenged α11^−/−^ mice have an incisor phenotype, causing malnutrition and increased mortality (110).

α11 mice fed regular laboratory chow are dwarfed, display increased mortality and suffer from malnutrition. Despite the abundant expression of α11 in perichondrial cells, α11 expression did not correlate with any cartilage growth defects. Instead, the malnutrition and smaller size observed in α11^−/−^ mice appears to correlate with the tooth phenotype. The phenotype observed in the α11 knockout mice is highly selective for the incisors, which, unlike the molars, erupt continuously in rodents. Whereas α11 appears to be expressed in the follicle mesenchyme of both molars and incisors, in the adult dentition it is mainly expressed in the incisors, which probably explains the restriction of the tooth phenotype to the incisors. The periodontal ligament has been shown to play a central role during rodent incisor eruption, but the exact nature of this role has been controversial. According to one school of thought, periodontal ligament fibroblasts migrate occlusally through the periodontal ligament space and create the tractional force that pulls the tooth toward the surface of the oral mucosa (14), whilst another school of thought maintains that this eruptive force is provided by the hydrostatic tissue pressure within the vascular tissue of the periodontal ligament (25). Many existing mutations described for the tooth affect the neural crest-derived cells and disturb tooth morphogenesis as a result, but relatively few of these selectively affect tooth eruption.

Until now there has been a lack of good genetic models for studying tooth eruption. We believe that the integrin α11-deficient mouse now offers one such model. *In-vitro* analysis of α11^−/−^ cells is a powerful tool for corroborating the *in-vivo* α11^−/−^ periodontal ligament phenotype. We have previously demonstrated that inactivation of α11β1 in mouse embryonic fibroblasts expressing α1β1, α2β1 and α11β1 increases cell migration on collagen I (112), presumably because of a lower strength of binding to collagen, offering less of a restraint on migration, but also leading to less tractional force generation. In the absence of α11β1, periodontal ligament cells bind periodontal ligament collagen with lower affinity. We predict that this changed interaction with collagen fibrils disturbs the tractional forces being developed, in turn contributing to the disturbed tissue homeostasis. The mutant incisor periodontal ligament in α11^−/−^ mice is characterized by a distinctly increased thickness, the presence of a wider acellular cementum and an increased number of epithelial cell rests of Malassez. The increased width of the cementum layer is probably related to a decreased eruption rate (13). Maintaining the correct width of the periodontal ligament is an essential function of the periodontal fibroblast and this regulation is obviously lost in the absence of periodontal ligament fibroblast α11β1. The present data thus identify α11β1 in periodontal ligament fibroblasts as a central regulator of periodontal ligament width. As judged by Sirius red staining, collagen accumulates in the mutant periodontal ligament. Integrins are known to regulate both collagen and matrix metalloproteinase synthesis (114, 118), and we attempted to determine whether increased synthesis was the mechanism behind this collagen accumulation, but quantification by biochemical methods was not possible because of the small amount of tissue. The numbers of periodontal ligament fibroblasts that can be isolated from the mouse incisor periodontal ligament are too low to allow analysis of collagen in primary cells. Immortalization of this cell population from wild-type and knockout animals may allow such an analysis in the future. In the case of the matrix metalloproteinases, we predict that matrix metalloproteinase activity would be reduced in the knockout mouse, but because the phenotype is only manifested postnatally, when ossification has occurred, sectioning of the periodontal ligament tissue involves a lengthy decalcification scheme, preventing reliable *in-situ* zymography to detect changes in matrix metalloproteinase activity. Interestingly, analysis of matrix metalloproteinases from mouse embryonic fibroblasts cultured inside a collagen gel revealed reduced induction of matrix metalloproteinase-13 and matrix metalloproteinase-14 in α11^−/−^ cells. Similar analysis of periodontal ligament tissue showed increased levels of matrix metalloproteinase-9 mRNA and reduced levels of matrix metalloproteinase-14 mRNA in the mutant periodontal ligament tissue (110). In summary, the exact mechanisms whereby α11 deficiency leads to a change in collagen turnover remain to be clarified, but they appear to involve changes in matrix metalloproteinase levels, leading to altered matrix turnover in the absence of integrin α11.

In summary, our data support a role for α11β1 in tooth eruption. We predict that α11β1 is needed on the surface of incisor fibroblasts to generate the tractional force needed for tooth eruption. Consistent with this model, the reduced capacity of periodontal ligament fibroblasts to adhere to and remodel the collagen matrix *in vitro* fits well with the phenotype of reduced tooth eruption observed *in vivo*. Given that α11β1 is needed in the periodontal ligament of the mouse incisor raises the possibility that this integrin also plays a role in periodontal ligament homeostasis in the human periodontal ligament. Very little is known at this stage about the role of α11β1 during human odontogenesis or about its role in the healthy and periodontitis-affected periodontal ligament. We predict that defects in the linkage between the surface of periodontal ligament fibroblasts and the collagen fibrils might predispose the subject to inflammatory conditions such as periodontitis. Although we did not see any evidence of inflammation in the periodontal ligament of α11 mutant mice (110), the potential importance of α11β1 for human periodontium integrity warrants further investigation of this subject matter (8). In addition to the incisor-eruption defect, the levels of systemic insulin-like growth factor-1 are reduced in α11^−/−^ mice (21), implying stromal α11-dependent regulation of the endocrine system. In what type of stromal cells, and in which endocrine organ this takes place, is currently unclear.

## Integrins and stem cells

At the time of writing this review there was limited information on the integrin repertoire of periodontal ligament progenitor and stem cells. It is therefore currently unknown if incisor and molar periodontal ligament stem cells have different integrin repertoires. The three stem-cell populations that are most interesting for future engineering in periodontal regeneration are mesenchymal stem cells isolated from the periodontal ligament, stem cells from exfoliated deciduous teeth and induced pluripotent stem cells (55). Whereas data on differentiation capacity and implantation effects with periodontal ligament stem cells exist and look promising, currently less is known about the feasibility of using induced pluripotent stem cells (36).

From a fundamental basic research point of view it would be interesting to characterize cell-adhesive events during culture, seeding onto membranes and grafting of stem cells into the injured (diseased) periodontal ligament tissue. From a therapeutic point of view, it would also be important to characterize the dynamic nature of the changing integrin repertoire of these cells during the clinical procedure. One can envision that cell culture of stem cells changes the integrin repertoire. This could be taken advantage of so that the scaffold used has a ligand composition matching the integrin repertoire of the cells to be grafted. It is also possible that culture of cells in a three-dimensional matrix might ‘normalize’ the integrin repertoire to a more *in-vivo-*like repertoire, possibly to enhance grafting into the tissue. We foresee that the area of cell-matrix integrin research in the context of stem cell-based therapeutic applications will play an increasingly important role.

## Summary

Collagen I can self-assemble *in vitro*, but in the periodontal ligament a highly organized pattern of collagen organization exists and we predict that periodontal ligament fibroblast integrins assist in this assembly (i.e. what we call the organizing role of integrins). The organizing role is important in order to maintain the homeostasis of the periodontal ligament tissue with a high turnover rate of collagen. To maintain/restore this organized structure during injury and in pathological situations we believe that it is essential to reorganize the newly synthesized periodontal ligament tissue and, in this respect, facilitate the return to normal tissue architecture. Also, integrins might play important roles in these events by activating transforming growth factor-beta, regulating matrix metalloproteinases and orchestrating the physical reorganization of the matrix.
